# Chemical Genetics Reveals an RGS/G-Protein Role in the Action of a Compound

**DOI:** 10.1371/journal.pgen.0020057

**Published:** 2006-04-21

**Authors:** Kevin Fitzgerald, Svetlana Tertyshnikova, Lisa Moore, Lynn Bjerke, Ben Burley, Jian Cao, Pamela Carroll, Robert Choy, Steve Doberstein, Yves Dubaquie, Yvonne Franke, Jenny Kopczynski, Hendrik Korswagen, Stanley R Krystek, Nicholas J Lodge, Ronald Plasterk, John Starrett, Terry Stouch, George Thalody, Honey Wayne, Alexander van der Linden, Yongmei Zhang, Stephen G Walker, Mark Cockett, Judi Wardwell-Swanson, Petra Ross-Macdonald, Rachel M Kindt

**Affiliations:** 1 Bristol-Myers Squibb Pharmaceutical Research Institute, Pennington, New Jersey, United States of America; 2 Exelixis Incorporated, South San Francisco, California, United States of America; 3 Hubrecht Laboratory, Centre for Biomedical Genetics, Utrecht, Netherlands; Huntsman Cancer Institute, United States of America

## Abstract

We report here on a chemical genetic screen designed to address the mechanism of action of a small molecule. Small molecules that were active in models of urinary incontinence were tested on the nematode *Caenorhabditis elegans,* and the resulting phenotypes were used as readouts in a genetic screen to identify possible molecular targets. The mutations giving resistance to compound were found to affect members of the RGS protein/G-protein complex. Studies in mammalian systems confirmed that the small molecules inhibit muscarinic G-protein coupled receptor (GPCR) signaling involving G-αq (G-protein alpha subunit). Our studies suggest that the small molecules act at the level of the RGS/G-αq signaling complex, and define new mutations in both RGS and G-αq, including a unique hypo-adapation allele of G-αq. These findings suggest that therapeutics targeted to downstream components of GPCR signaling may be effective for treatment of diseases involving inappropriate receptor activation.

## Synopsis

The authors have utilized *Caenorhabditis elegans,* and yeast genetics, combined with mammalian tissue and cell culture experiments to investigate the mechanism of action of a unique set of small molecules. These molecules are active in tissue models of urinary incontinence and allow for increased bladder filling. In the course of studying sensitivity and resistance to these compounds, Fitzgerald et al. uncovered novel alleles of RGS and Gq proteins. Further characterization of one such allele identified that its action conferred a hypo-adaptive phenotype on yeast during pheromone signaling assays. Their data as a whole indicate that these small molecules are able to diminish signaling from G-protein coupled receptors (GPCR) downstream of the receptors themselves. Since GPCR signaling is very important in many diseases in humans, the novel mechanism of these compounds may offer new ways to treat human disease.

## Introduction

Urinary incontinence (UI) is an increasing medical problem in ageing populations. Affecting more than 12 million afflicted people in the US alone, UI is a frequent cause of confinement and lifestyle modification [1]. UI is defined as the involuntary loss of urine, and may result from a number of causes including the improper control of detrusor activity or compromised urethral function. UI can also occur as a complication of other diseases such as Parkinson disease, multiple sclerosis, and bladder infections, indicating that there are both muscular and neuronal components of the disease.

Current treatments for UI rely on antagonism of G-protein coupled receptors (GPCRs) of the muscarinic acetylcholine receptor class [2]. The signal transduction pathways downstream of muscarinic GPCRs are responsible for bladder muscle cell contractility, and antagonists of these receptors allow for greater bladder filling. While muscarinic GPCR antagonists are generally safe, they have unwanted side effects due to the broad tissue expression of their targets [3–5]. GPCRs are the most successful class of targets for disease states including hypertension, diabetes, obesity, depression, osteoporosis, and inflammation. In fact, more than half of currently marketed drugs for the condition act as modulators of this protein class [6,7]. Methods to modulate other signaling nodes downstream of GPCRs may hold potential for safer and more efficacious therapies.

Heterotrimeric G-proteins are the proximal signaling partners downstream of GPCRs. Binding of acetylcholine to the muscarinic GPCRs results in the exchange of GDP for GTP on the G-protein α subunit (G-αq). This activation event allows dissociation of G-αq from the G-β/γ heterodimer. The dissociated G-protein subunits then mediate separate cellular responses through their interactions with enzymes, channels, kinase cascades, and intracellular second messengers [8–10]. In smooth muscle cells, activation of G-αq results in protein kinase C (PKC)-dependent calcium mobilization and subsequent muscle contraction. Following GTP hydrolysis on G-α, the heterotrimeric G-protein complex reforms and signaling is terminated. G-protein function is under the strict control of factors such as the regulators of G-protein signaling (RGS) proteins. RGS proteins were first identified as potent negative regulators of GPCR signaling in yeast [11], and are now known to act as GTPase activating proteins (GAPs) in all eukaryotic systems. RGS proteins bind directly to the G-α subunit and enhance the rate of GTP hydrolysis, thereby shortening the lifetime of the dissociated, active G-protein species and curtailing GPCR signaling [12]. At least 24 mammalian proteins contain a common “RGS core domain” [13]. Interestingly, many RGS proteins have been shown to have spatially restricted expression patterns, suggesting that they may allow tissue-specific control of ubiquitous G-proteins [14,15].

In this paper we describe mechanism-of-action studies with small molecules that were originally identified by their activity in an ex vivo bladder contraction assay. These effects are mediated by a heretofore unknown molecular mechanism [16]. Notably, in vivo studies using a selected example (BMS-195270) revealed that this small molecule displayed marked tissue specificity, inhibiting bladder contractility at doses that did not significantly affect blood pressure or heart rate [16] (unpublished data). To define the pathway of action of these small molecules, we have used genetic screens in Caenorhabditis elegans coupled with biochemical assays in mammalian systems. We demonstrate that these small molecules likely act at the intersection of RGS and G-αq proteins, resulting in the downregulation of GPCR signaling, reduced calcium fluxes, and reduced muscle contraction. In addition, we have uncovered novel mutations in RGS and G-αq proteins, including the first hypo-adaptation allele of a G-αq protein. Identification of a novel set of compounds that function to limit signaling downstream of GPCRs, as well as a hypo-adapation allele of G-αq, has implications for the many diseases currently treated via direct modulation of these receptors.

## Results

### Small Molecules That Affect Mammalian Rat Bladder Muscle Contraction via an Unknown Mechanism

Several related small molecules (Figure 1) were identified as active in a screen for inhibition of carbachol-evoked tonicity of isolated rat bladder strips. The effects of BMS-195270 in an ex vivo rat whole bladder model are shown in Figure 2. Typical cystometric curves (bladder pressure plotted versus infused volume) for BMS-195270–treated or vehicle-treated bladders are shown in Figure 2A and Figure 2B. Incubation of bladders in BMS-195270 produced a dramatic reduction in developed pressure at infusion volumes of 0.2–1.3 mls (Figure 2A; *n* = 5, *p* < 0.05) relative to the vehicle-only treatments over the same filling volume range (Figure 2B; *n* = 12 bladders). BMS-195270 treatment was also found to inhibit “spontaneous” contractions (Figure 2C and 2D). The finding that BMS-195270 was able to increase the filling capacity and reduce spontaneous contractions in this ex vivo model underscored the potential utility of this class of small molecules in treating UI resulting from overactive bladder contractions. Assays of structurally related small molecules revealed two small-molecule pairs that each contain one active and one inactive small molecule (BMS-192364/BMS-192365 and BMS-195270/BMS-195243; Figure 1A–1D, [16]). The mechanism of this bladder-specific activity, however, was not known.

**Figure 1:**
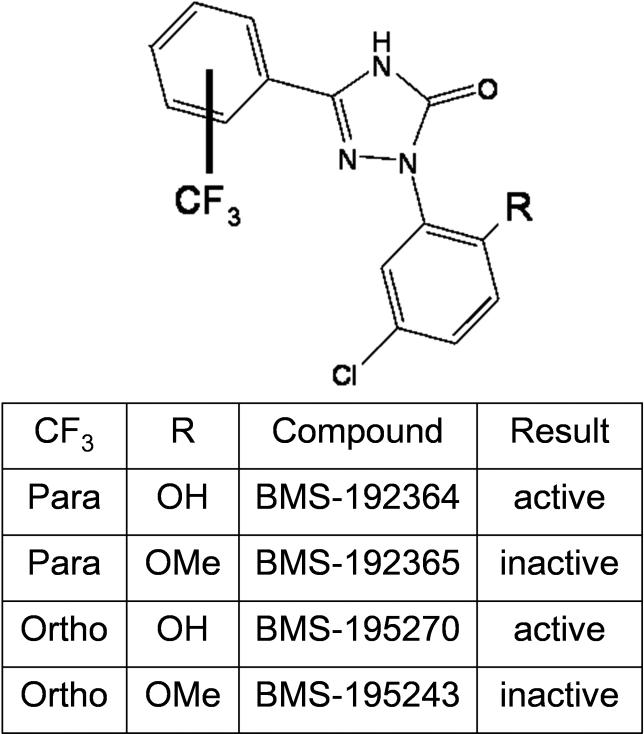
Structure and Activity of BMS Small Molecules The effect of each compound on inhibition of carbachol-evoked tonicity of isolated rat bladder strips is shown in the table.

**Figure 2:**
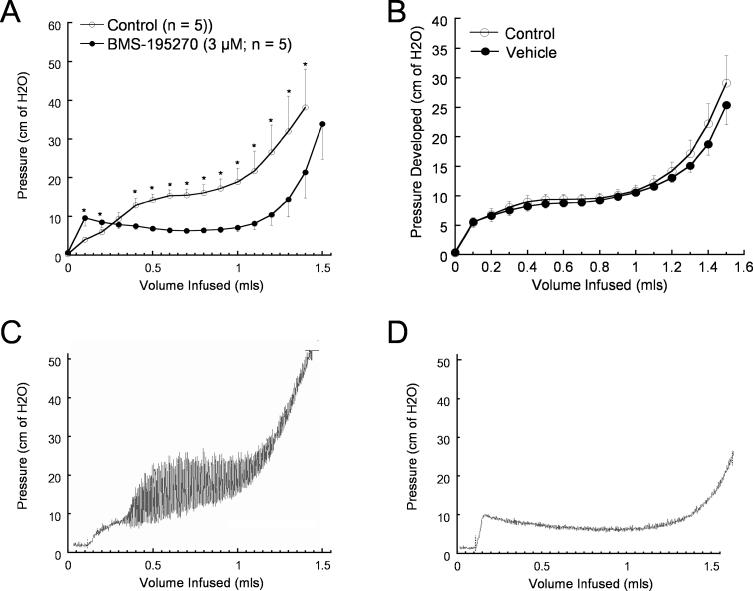
Effect of BMS Small Molecules in Ex Vivo Whole-Bladder Assays Bladder pressure was monitored during saline infusion in the presence of BMS-195270 or vehicle. A typical cystometric curve shows three phases: a small rapid rise in intravesical pressure followed by a plateau phase and then a final sharp increase in pressure. (A) Bladder pressure following treatment with BMS-195270 (3 μM; *n* = 5 bladders). The average pressure at discrete infusion volumes is shown. Significant differences in pressure relative to control (*p* < 0.05) are indicated by asterisks. (B) Bladder pressure following treatment with vehicle (*n* = 12 bladders). The average pressure at discrete infusion volumes is shown. No difference in pressure relative to control was seen. (C) Representative trace from vehicle-treated bladder, showing magnitude of spontaneous contractions developed during saline infusion. (D) Representative trace from bladder treated with BMS-195270 (3 μM), showing reduction in frequency and magnitude of spontaneous contractions developed during saline infusion.

**Table 1:**
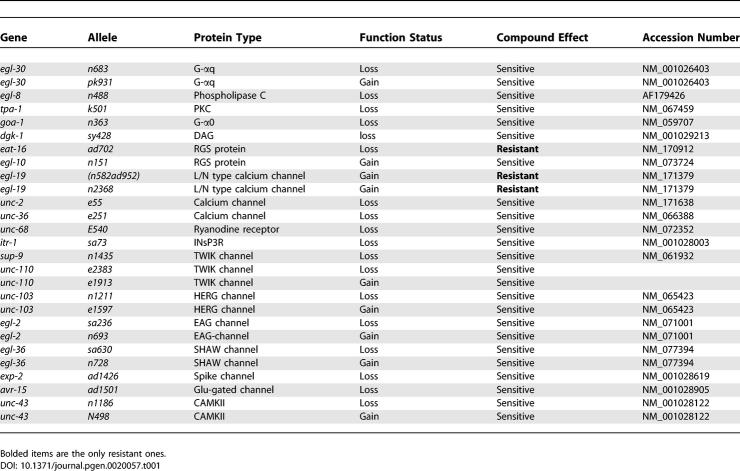
Description of C. elegans Mutant Alleles Used

### A Chemical Genetic Approach in C. elegans to Identify Candidate Targets

We next utilized C. elegans to elucidate the molecular pathway affected by these small molecules. First, the active small molecules BMS-192364 and BMS-195270 were applied to wild-type adult *C. elegans,* and the resulting phenotypes observed (see Materials and Methods). Note that treatment of C. elegans with small molecules typically requires a higher concentration than cell-based assays, due to the worms' relatively impermeable cuticle.

Treatment of adult worms with 0.3 mM BMS-192364 resulted in a bloated egg-laying defective (Egl-d) phenotype that included retention of fertilized late-stage eggs (Figure 3A). This Egl-d phenotype indicates defective neuromuscular function in the egg-laying process. Treatment with BMS-192364 is dose-responsive, with a cumulative total of >90% of animals ultimately displaying an Egl-d phenotype (Figure 3B). Treatment of adult worms with BMS-195270 at 2.8 mM resulted in a similar Egl-d phenotype, as well as slowed or arrested pharyngeal pumping (Eat), and uncoordinated motion (Unc). These phenotypes also indicate a defect in normal neuromuscular signals. The pumping and movement defects were apparent within two hours of treatment, and the Egl-d phenotype was noted within 12 hours of treatment and maximal after 24 hours, consistent with an acute effect on the neuromuscular system. Structurally related small molecules that lacked the bladder-relaxing activity, such as BMS-192365, did not cause the Egl-d, Eat, or Unc phenotypes under any treatment conditions (unpublished data). This observation suggested that the structure–activity relationship as determined in the mammalian systems held true in *C. elegans,* and that the worm phenotypes corresponded to the therapeutic activity of the compounds. BMS-192364 was the more potent of the two active molecules in the egg-laying assays, and subsequent analyses in C. elegans used this compound.

**Figure 3:**
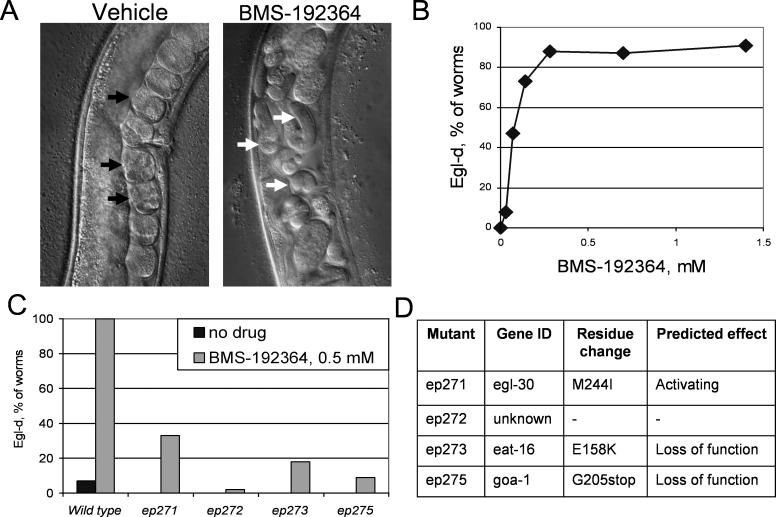
Effect of Small Molecules on Wild-Type and Mutant C. elegans (A) The gonad/vulval region of wild-type worms is shown. In the left panel, black arrows indicate the normal, organized array of early stage eggs. The right panel shows a worm treated with BMS-192364 at 0.3 mM. The white arrows indicate late stage eggs that have been retained in the gonad. (B) Dose-response curve for BMS-192364, showing effect on egg laying in C. elegans. The percentage of worms displaying an Egl-d phenotype was determined by counting the number of “commas” contained within the animal. (C) Quantification of the Egl-d phenotype in four C. elegans mutant strains—*ep271, ep272, ep273,* and *ep275—*that were identified in a screen for resistance to the small molecule. Black bars, no treatment. Grey bars, worms treated with BMS-192364 at 0.4 mM. (D) Table showing identity of the affected gene in *C. elegans–*resistant mutant strains, the amino acid changes, and predicted effect on protein function.

To identify potential molecular targets or target pathway components for the small molecules, we first took a candidate-gene approach. The G-protein mediated pathways involved in egg laying have been extensively characterized in *C. elegans.* We tested BMS-192364 on 27 different worm strains, each carrying a mutation in pathways implicated in egg laying (Table 1). Of the 27 strains tested, three exhibited at least partial resistance to the Egl-d phenotype induced by BMS-192364. (Resistance was defined as no more than 15% of animals exhibiting an Egl-d phenotype in the presence of a small-molecule dose which renders >90% of wild-type animals Egl-d). The three resistant strains carried either the *eat-16(ad702),* the *egl-19(n582ad952),* or the *egl-19(n2361)* mutation.

Interestingly, the mutation giving rise to the strongest resistance phenotype, *eat-16(ad702),* disrupts an mRNA splice acceptor site and leads to truncation of an RGS protein. The EAT-16 RGS protein would normally downregulate G-protein signaling, an activity consistent with the phenotype of the small molecule [17]. The *nIS51(egl-10+)* allele phenotypically resembles the *eat-16(ad702)* allele (it overexpresses the EGL-10 RGS protein, which acts in an antagonistic pathway). However while the *eat-16(ad702)* strain was resistant to BMS-192364, a strain carrying the *egl-10(nIS51)* allele showed no resistance. Thus the resistance of the *eat-16(ad702)* strain seems closely linked to EAT-16 signaling status. In addition, the lack of resistance or hypersensitivity exhibited by strains carrying mutations in *egl-8, dgk-1, tpa-1,* or *unc-68* (encoding phospholipase C, diacylglycerol kinase, PKC, and the Ryanodine receptor; Table 1) indicated that BMS-192364 was most likely acting upstream of or parallel to these genes' products. Taken together, the candidate-gene results suggested a target function in the area of the neurotransmitter/GPCR/G-protein complex. The resistance exhibited by two strains carrying gain-of-function mutations in *egl-19* [18] also indicated that calcium channels could be the target of BMS-192364.

Genetic screens designed to identify both dominant and recessive mutations have been used successfully to identify components of pathways affected by small molecules [19,20]. In parallel to the candidate gene analysis, we also decided to carry out an unbiased genetic screen based on the robust Egl-d phenotype, looking for resistance to the small molecule BMS-192364. The candidate gene analysis indicated that such a screen should yield resistant mutants. 150,000 EMS-mutagenized genomes were generated and screened, resulting in the identification of four resistant mutants (Figure 3 C). In all cases these mutants were egg-laying constitutive (Egl-c) in the absence of treatment, and both the Egl-c phenotype and the drug resistance were dominant or semidominant. All BMS-192364–resistant mutants were also found to be cross-resistant to the phenotypes induced by BMS-195270 (unpublished data).

### Dominant *C. elegans–*Resistant Mutations Affect G-Protein Pathway Components

The mutations present in the three resistant strains *ep271, ep273,* and *ep275* were mapped to defined chromosomal regions using recombinant single nucleotide polymorphism (SNP) techniques [21,22]. Genes in these regions that were known to confer an Egl-c phenotype were obvious candidates. Linkage tests to known alleles, and direct sequence analysis, showed that three of the four mutated genes were allelic to the known genes *egl-30, eat-16,* and *goa-1* (Figure 3D). The identity of the gene affected in the fourth resistant strain, *ep272,* remains unknown.

Strain *ep271* carried a mutation in *egl-30,* which encodes the worm ortholog of the G-αq subunit and is required for egg laying [17,23]. Based on the Egl-c mutant phenotype observed, the mutation found in the *egl-30(ep271)* allele, M244I, is predicted to constitutively activate G-αq signaling. Methionine-244 of EGL-30 is conserved in many G-proteins that are regulated by an RGS domain–containing protein (Figure 4A).

**Figure 4:**
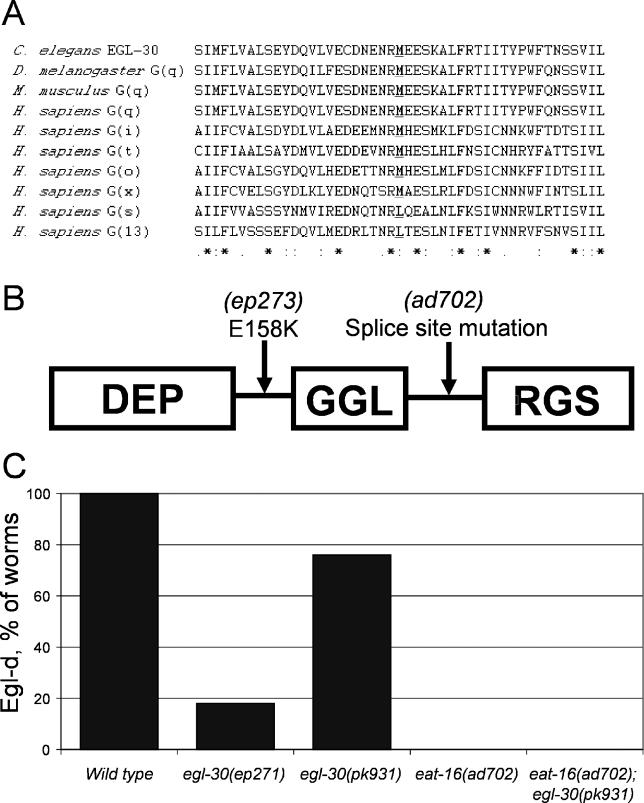
Protein Mutants Identified and Epistasis Tests (A) Alignment of a region of EGL-30 containing the M244I amino acid substitution with other G-proteins. The conserved methionine residue that is mutated to isoleucine in the *egl-30(ep271)* allele is indicated in bold text and underlined. (B) Diagram showing the domain structure of the EAT-16 protein with relative locations of the E158K substitution found in the *eat-16(ep273)* allele, and the splice site mutation found in *eat16(ad702)* strains. (C) Genetic interactions between mutations in G-αq and RGS. The G-αq/*egl-30(ep271)* and RGS*/eat-16(ad702)* mutations confer resistance to BMS-192364. The sensitivity of a G-αq/*egl-30(pk931)* strain to BMS-192364 is abrogated in the presence of the *eat-16(ad702)* mutation.

Strain *ep273* carried a mutation in *eat-16,* a member of the conserved RGS protein family [17]. EAT-16 has been shown to directly interact with and negatively regulate signaling via the G-αq protein EGL-30 [17,24,25]. The *eat-16(ep273)* allele was found to generate a nonconservative change, E158K, at a location between the DEP and GGL regions of the protein (Figure 4B). The Egl-c phenotype of the *eat-16(ep273)* mutant is similar to known loss-of-function *eat-16* mutants [17,24].

Strain *ep275* carried a mutation in *goa-1* [17,26], which encodes the worm G-αo protein and acts to negatively regulate egg laying, most likely acting in parallel to the *egl-30*/*eat-16* pathway [23,27]. The *goa-1(ep275)* allele was found to contain a stop codon that truncates the GOA-1 protein at amino acid 205, and is predicted to produce an inactive protein (Figure 3D).

The results of the genetic screen were striking. All three of the cloned resistant alleles (two dominant and one semidominant) affect proteins that are members of G-α/RGS complexes. This result, combined with the candidate gene analysis (Table 1), indicated that the small molecules were most likely acting on the signaling pathway at the level of the G-protein complexes. To summarize the genetic results, we observed two strongly compound resistant loss-of-function *(lf)* alleles of *eat-16,* one resistant and one sensitive gain-of-function *(gf)* allele in *egl-30,* and one resistant and one sensitive *(lf)* allele in *goa-1,* and finally two resistant *(gf)* alleles of *egl-19,* which encodes an L-type calcium channel [18]. The location of these gene products in a canonical signaling pathway is shown in Figure 5. In both the candidate gene analysis and the genetic screen, the strongest resistance was displayed by *eat-16 (lf)* alleles. The differential resistance of the *egl-30(ep271)* and *egl-30(pk931)* strains (Figure 4C) was quite surprising, as without compound treatment they display an identical Egl-c *(gf)* phenotype. This might indicate that these alleles have obtained their gain-of-function properties through different mechanisms: the *egl-30(pk931)* allele was originally identified as a suppressor of *gpb-2* overexpression phenotypes (RP, personal communication).

**Figure 5:**
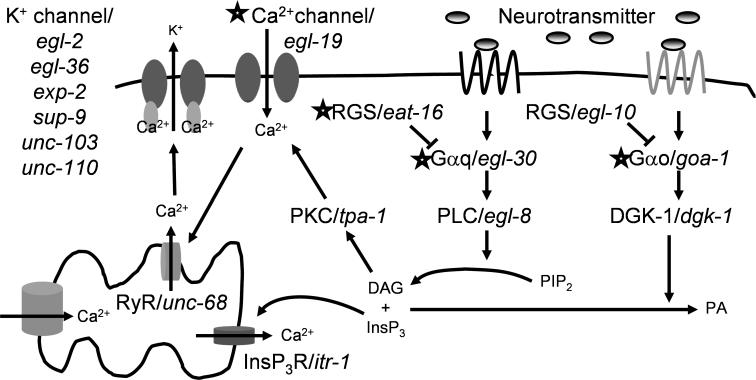
Analysis of Molecular Signaling Events Controlling Muscular Contraction in C. elegans The diagram shows the molecular components of the signaling pathways downstream of the muscarinic GPCRs in *C. elegans*. Alleles of genes encoding proteins in the pathway were tested for their effect on the Egl-d phenotype caused by treatment with BMS-192364. For the pathway members indicated by stars, certain alleles altered the response to treatment with BMS-192364. Specifically, resistance to the Egl-d phenotype was conferred by two gain-of-function alleles of *egl-19* and one of *egl-30,* and by two loss-of-function alleles of *eat-16* and one of *goa-1*.

### Confirmation of G-Protein Involvement Using a Mammalian System

The functional roles of the C. elegans modifier genes pointed us toward a small-molecule mechanism involving calcium signaling via G-proteins (Figure 5). To validate and extend this model in mammalian cells, the small molecules were evaluated for their effects on muscarinic GPCR-mediated calcium release in mammalian cells. Both BMS-192364 and BMS-195270 inhibited the response of HEK293 cells to the muscarinic agonist carbachol (Figure 6A and 6B; the EC50 for BMS-192364 was 9 μM, and for BMS-195270 was 2 μM). Experiments with muscarinic receptor subtype-specific inhibitors indicated that the majority of the carbachol-evoked signal was via muscarinic receptor type 3 (M3), with a small amount via M2 (unpublished data). The degree of inhibition observed with BMS-192364 and BMS-195270 was similar to that seen with known pan-muscarinic receptor inhibitors. The ability of the small molecules to inhibit calcium fluxes downstream of muscarinic receptor activation was entirely consistent with our observations in mammalian bladder tissue and C. elegans.

**Figure 6:**
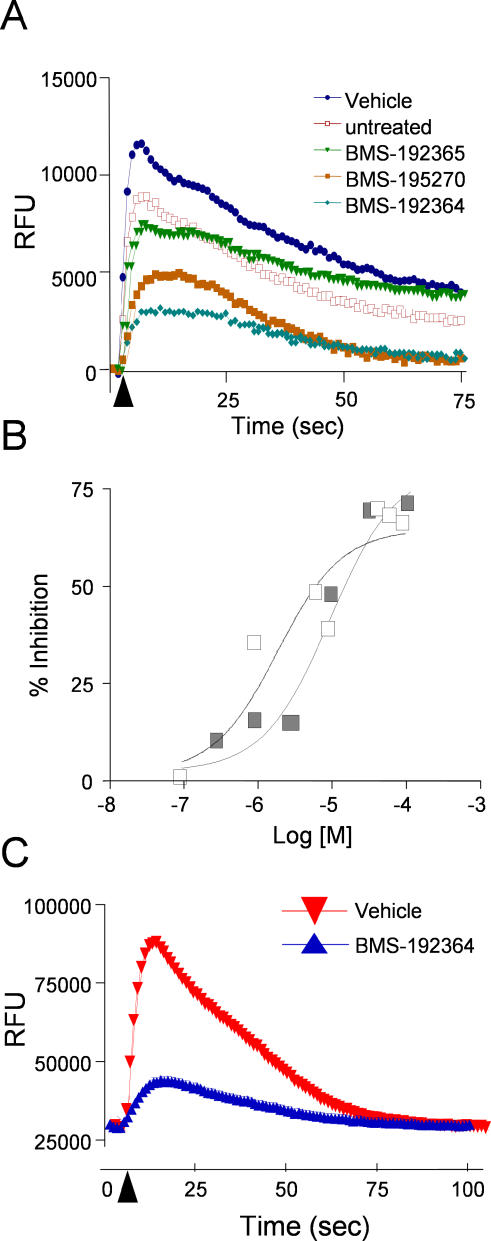
Effect of Compounds on Agonist-Induced Calcium Flux (A) The graph displays fluorescence intensity measurements for HEK293 cells preloaded with Fluo-4. Five baseline fluorescence measurements were taken prior to the injection of the muscarinic GPCR agonist carbachol. The timing of agonist addition is indicated by a black arrowhead. Measurements were performed in the presence of vehicle or the BMS small molecules indicated, at 100 μM. (B) Dose-response analysis for the effect of compounds on carbachol-stimulated calcium flux. White squares, BMS-195270; EC_50_ 2 μM. Black squares, BMS-192364; EC_50_ 9 μM. (C) The graph displays fluorescence intensity measurements for primary smooth muscle cells preloaded with Fluo-4. Five baseline fluorescence measurements were taken prior to the injection of the GPCR agonist histamine. The timing of agonist addition is indicated by a black arrowhead. Measurements were performed in the presence of vehicle or BMS-192364 (100 μM).

Utilizing a primary bladder smooth muscle cell line with Histamine as a receptor agonist, we also observed inhibition of calcium flux by BMS-192364 (Figure 6C) and BMS-195270 (unpublished data). Our genetic results had suggested that the compounds acted downstream of muscarinic receptors. To further investigate this, we carried out competitive binding assays with BMS-192364 and the radio-labeled muscarinic ligand N-methylscopolamine. We observed no effect on radio-ligand binding to the muscarinic receptor types M1–5 [28,29] using BMS-192364 at concentrations of 10μM. Similar radio-ligand binding experiments with the histamine receptors H1–4 were also negative (unpublished data) [30–32]. While we can not rule out the existence of a previously undescribed allosteric site shared by the two GPCR types, from these results it is unlikely that BMS-192364 and related small molecules act directly on muscarinic or histamine receptors. Additional BMS compound/radio-ligand binding assays run on adrenergic, angiotensin, bradykinin, cannabinoid, dopamine, endothelin, neuropeptide Y, nicotinic, serotonin, and other GPCRs were also negative.

The mammalian N- and L-type calcium channels were also of interest as potential targets, due to the partial resistance exhibited by two *egl-19(gf)* alleles in C. elegans (Table 1). To address the possibility that BMS-192364 and BMS-195270 were acting directly on the channels, we repeated the carbachol-stimulated Ca-flux assays, but pretreated cells with a high concentration of the calcium channel blocker niguldipine [33]. We reasoned that if BMS-195270 were acting directly on calcium channels, by preblocking those channels with niguldipine, we should observe no further effect of BMS-195270 on calcium flux. We observed that while pretreatment with niguldipine slightly dampened carbachol-induced calcium flux, BMS-195270 treatment was clearly additive to this effect (Figure 7A). This additive response indicated that BMS-195270 retained inhibitory activity even when endogenous calcium channels were inactivated. In addition to the cellular assays, competitive binding assays were performed using radio-labeled diltiazem (L-type ligand) [30] or ω-conotoxin (N-type ligand) [34]. BMS-192364 at 10μM had no effect on binding of either radio-ligand, suggesting that the L- and N-type channels are not the direct target of BMS-192364.

**Figure 7:**
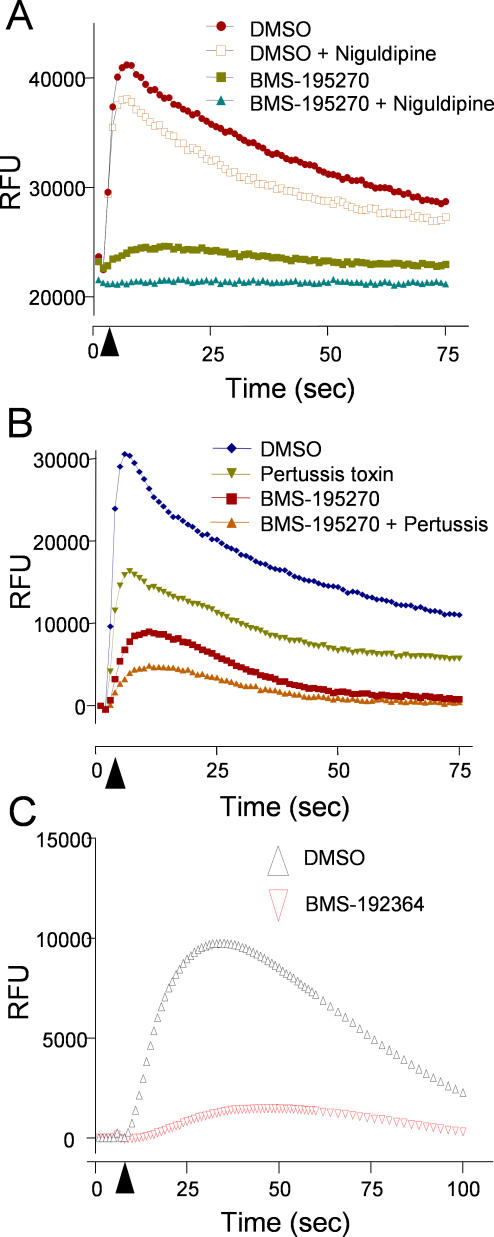
Effect of BMS-192364 in Combination with Other Modulators of Calcium Signaling The graphs display fluorescence intensity measurements for HEK293 cells preloaded with Fluo-4 then stimulated with the muscarinic GPCR agonist carbachol at 100 μM. Five baseline fluorescence measurements were taken prior to the injection of carbachol. Where indicated, BMS-195270 or BMS-192364 (100 μM) were added 15 min prior to the carbachol stimulation. The timing of carbachol addition is indicated by a black arrowhead. (A) Where indicated, cells were pre-incubated for 15 min with the calcium channel blocker niguldipine (100 μM). (B) Where indicated, cells were pre-incubated for 24 h with the G-protein antagonist pertussis toxin (150 ng/ml). (C) The treated cells are overexpressing the G-αq mutant allele G188S, which is known to be insensitive to RGS GAP activity.

Our genetic analysis for resistance to BMS-192364 had identified multiple alleles affecting the RGS protein EAT-16 and single alleles for G-αq *(egl-30)* and G-α0 *(goa-1).* The genetic data also indicated that the targeted process was likely upstream or parallel to PKC *(tpa-1),* PLC *(egl-8),* DGK *(dgk-1),* and the RyR *(unc-68),* as mutations in these genes did not affect sensitivity to BMS-195270 (Table 1). Clearly a comprehensive analysis of potential branched effecter pathways needs to be carried out. Consistent with these genetic observations, pretreatment of HEK293 cells with inhibitors of the ryanodine receptor (ryanodine), PI3-kinase (wortmanin), or PLC (U73122) did not affect inhibition of carbachol-induced calcium flux by BMS-195270 (unpublished data). Therefore, we next examined the G-protein/RGS complex more closely.

In *C. elegans,* the RGS protein EAT-16 has been shown to interact with both G-αq/EGL-30 and G-α0/GOA-1, in different complexes. Therefore, we investigated which G-α subunit was involved in the BMS-195270 activity seen in HEK293 cells. Given that the muscarinic receptor M3 (a G-αq–coupled receptor) was responsible for the majority of the carbachol-induced signal, G-αq was the most likely candidate. We tested the effects of BMS-195270 in combination with pertussis toxin, which is a known blocker of G-αo/i and G-αs signaling but does not affect G-αq [35]. Pertussis toxin has been utilized extensively to differentiate G-α signals. Pertussis toxin reduced the peak calcium flux evoked by carbachol stimulation of HEK293 cells, but the effect of pertussis toxin was nearly additive with that of BMS-195270 (Figure 7B). This result indicated that BMS-195270 inhibits signaling downstream of muscarinic receptors, at least in part, by a G-αq-dependent mechanism. This observation was entirely consistent with the genetic analysis in *C. elegans,* but does not exclude the possibility that some BMS-195270 activity in mammalian cells might also be due to inhibition of another G-α subtype activity.

### Genetic Tests Differentiate between Models of Small-Molecule Action

The biochemical and genetic data collectively directed us toward a hypothesis that the BMS small molecules affect the RGS/G-protein complex. In simple, single-target models, the behavior of BMS-192364 and BMS-195270 is consistent with action as an RGS protein agonist, or as a G-α antagonist. In the former model, the small molecule would increase the rate at which RGS protein is able to stimulate GTP catalysis by G-α (RGS GAP activity) and thereby shorten the active period of G-protein signaling. In the latter model, the small molecule inhibits G-αq activity directly. The small molecule could affect the activation of G-αq or its ability to signal to downstream partners (Figure 8). A precedent for small-molecule effects on G-αq has been set by YM-254890 [36], which has been reported to inhibit the activity of G-αq. Genetic tests in a variety of systems were used to investigate these two single-target models.

**Figure 8:**
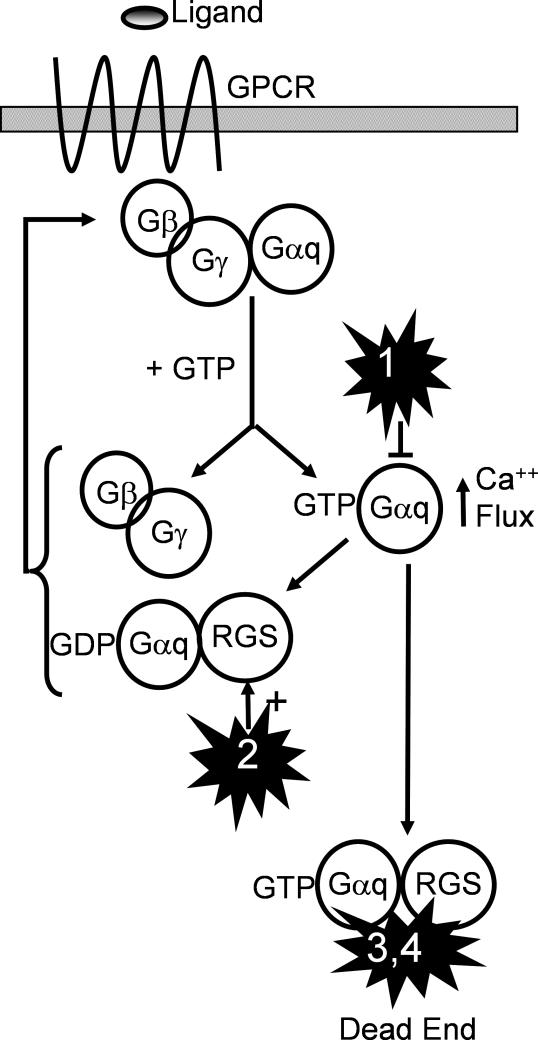
Models for Mechanism of Action Four models of small-molecule action are presented. In model 1, the small-molecule acts directly and uniquely as an antagonist of G-αq. In model 2 the small-molecule acts directly and uniquely as an agonist of the RGS protein's GAP activity. In models 3 and 4 (our preferred models), the small molecule interacts with both the RGS protein and G-αq, leading to an increased affinity of RGS for the complex and/or an “abortive” complex (failure of G-αq to recycle). All models lead to reduction of the GPCR signal through the activated G-αq.

### A Genetic Test of the RGS Agonist Model in Yeast

If the compounds act via the RGS protein, G-αq proteins that are resistant to RGS GAP activity should also exhibit resistance to the compound. In C. elegans the *egl-30 (ep271)* allele of G-αq, which contains the amino acid substitution M244I, confers resistance to compound BMS-192364. Therefore, we wished to determine whether this mutation also confers resistance to RGS GAP activity, as predicted by the first model.

Ideally, resistance to compound activity and to RGS GAP activity should be studied in the same genetic system. However, there are no C. elegans strains that carry bichemically proven RGS GAP–insensitive alleles of G-α, and there is no system for evaluating response to RGS GAP activity in *C. elegans.* Instead, RGS GAP activity is studied in the yeast *Saccharomyces cerevisiae,* which offers both a robust assay system and also the ability to substitute the mutant alleles under analysis [11,37]. (Unfortunately, the insolubility of compound BMS-192364 in yeast growth media prohibits direct evaluation of its action in yeast). The yeast RGS/G-α interaction is highly conserved with respect to higher eukaryotes, and the resistance of a mammalian G-α mutant protein to RGS GAP activity has been determined in this system [11,37]. Therefore, observations in yeast will be informative as to the properties of the *C. elegans egl-30(ep271)* allele.

RGS/G-α interactions in yeast are evaluated by treatment with the peptide ligand α–factor, which acts on the GPCR Ste2 initiating a signaling cascade that blocks cell division. This can easily be assayed as a halo of growth arrest around a point source of the ligand (Figure 9A) [11,38–42]. In the absence of the GAP activity of yeast RGS protein Sst2, this growth arrest can be achieved at lower ligand concentrations, hence larger halos are observed (Figure 9A). To test whether the EGL-30(M244I) substitution produced a G-α protein that was sensitive to RGS GAP activity, we constructed the equivalent mutation, M362I, in the yeast G-α protein Gpa1. The *gpa1-M362I* or wild-type *GPA1* alleles were then expressed from a plasmid [43] in the background of a yeast strain lacking both the RGS and wild-type G-α *(sst2 gpa1)*. When tested in the halo-formation assay, the *sst2 gpa1 (p-gpa1-M362I)* and the *sst2 gpa1 (p-GPA1)* strains produced the same diameter of halo, demonstrating that a functional G-α protein is produced from the *gpa1-M362I* allele (Figure 9A and 9B). The halo diameter was large, as expected in the absence of the Sst2 RGS function. When Sst2 function was returned to the two yeast strains (by expression of the *SST2* gene from a second plasmid), halo diameter was reduced to the same degree in the presence of the *gpa1-M362I* or the wild-type *GPA1* allele (Figure 9C and 9D). Thus, the yeast *gpa1-M362I* allele produces a functional G-α protein that has a normal response to the GAP activity of the RGS protein. Since the equivalent G-α allele in *C. elegans, egl-30(M244I),* is resistant to BMS-192364, a model in which the small molecule acts as an RGS protein agonist is unlikely.

**Figure 9:**
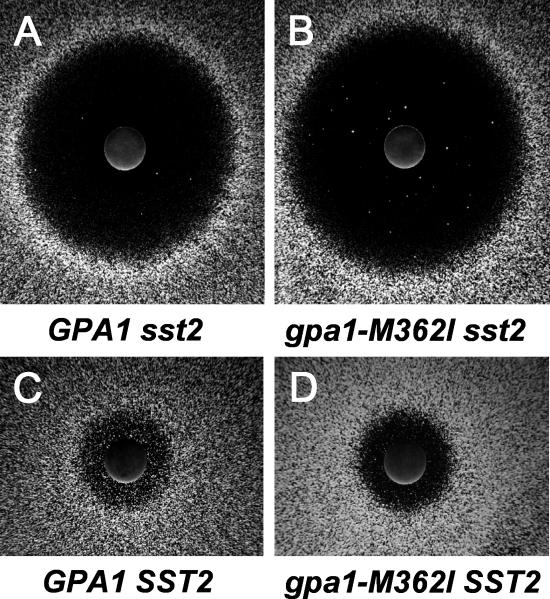
The Yeast *gpa1-M362I* Mutant Allele Causes a Hypo-Adaptation Phenotype Images show the growth of a monolayer of yeast cells around a paper disc containing alpha factor, the peptide ligand for the Ste3 GPCR. A zone of growth inhibition is visible as a “halo” around each disc. (A) Yeast strain contains a wild-type G-αq gene *(GPA1)* and has a chromosomal deletion of the *SST2* gene, encoding an RGS protein. (B) Yeast strain contains a wild-type G-αq gene *(GPA1)* and a chromosomal deletion of the *SST2* gene, but carries wild-type *SST2* on a plasmid. (C) Yeast strain contains a mutant G-αq gene *(gpa1-M362I)* and has a chromosomal deletion of the *SST2* gene, encoding an RGS protein. (D) Yeast strain contains a mutant G-αq gene *(gpa1-M362I)* and a chromosomal deletion of the *SST2* gene, but carries wild-type *SST2* on a plasmid.

In the yeast halo formation assay, one qualitative difference was apparent between the *p-gpa1-M362I* and the *p-GPA1* strains, regardless of their RGS status. The halos produced by the strain expressing Gpa1-M362I protein were less turbid (Figure 9B and 9D), indicating that growth was arrested for longer by a given dose of pheromone. The phenomenon of growth resumption in the continued presence of the α–factor ligand is called adaptation [42]; the *gpa1-M362I* allele is thus hypo-adaptive. Interestingly, the M362I mutation affects a region of Gpa1 where a number of hyper-adaptation alleles have also been described [42,44,45] (Figure 10). These mutations all affect amino acid residues on the surface that is known to interact with downstream effectors of G-α [46]. Thus the *C. elegans egl-30(M244I)* mutation is likely to confer resistance to compound by strengthening interactions with effectors, rather than by insensitivity to RGS GAP activity. This M/I substitution creates the first hypo-adaptation allele of G-α to be described.

**Figure 10:**
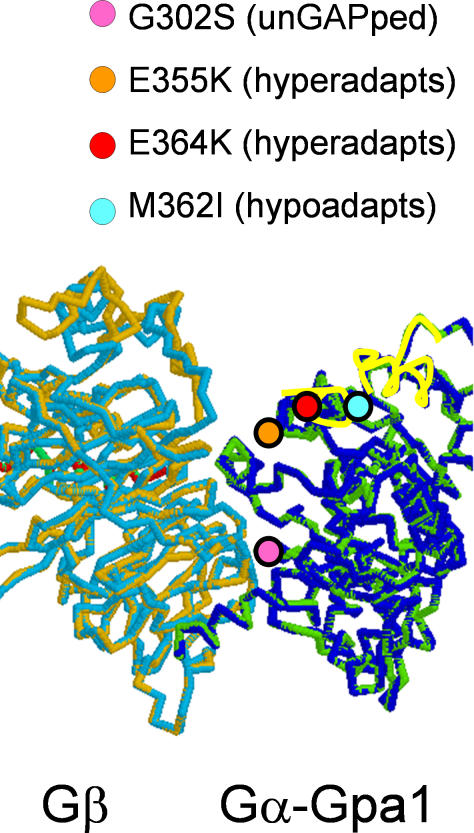
Amino Acid Substitutions in Mutants of the Yeast G-αq Protein Gpa1 A theoretical three-dimensional structure of the yeast Gpa1 G-α protein in complex with the Ste4 protein (G-β) is shown. The position of four amino acid substitutions with phenotypes of interest is indicated by circles. Two alpha helices are indicated by yellow highlighting of the protein backbone. In higher eukaryotes, these helices are considered to form the interface with G-αq downstream effector proteins. Three mutations affecting adaptation to mating pheromone lie on this face: E355K and E364K both hyper-adapt while the M362I allele described in this work is hypo-adaptive. For reference, the position of a mutation affecting sensitivity to RGS GAP activity, G302S, is also shown.

### A Genetic Test of the G-α Antagonist Model in *C. elegans*

The evidence presented so far suggested that the compound could be acting on the G protein, the RGS protein or potentially both. We devised a genetic test to attempt to help distinguish between these possibilities. The experiment took advantage of a putative activated *egl-30* mutant that is sensitive to the compound (unlike the egl-30(M244I) mutant identified in the resistance screen). The *egl-30(pk931)* strain has an Egl-c phenotype, indicative of constitutively activated G-αq signaling. The R210Q substitution in *egl-30(pk931)* affects a predicted key residue on the face of G-α that interacts with the RGS protein. While there is no direct proof that EGL-30(R210Q) is constitutively activated due to an insensitivity to RGS GAP activity, a mammalian G-αq mutation of the neighboring amino acid (Q209L) results in both constitutive activation and insensitivity to RGS GAP activity. The *egl-30(pk931)* strain was found to retain sensitivity to BMS-192364 (Figure 4C). The finding that different mutant alleles of the same gene have different sensitivities to the compound suggests that the G-α protein, EGL-30, may be directly involved in compound action. However, it cannot be ruled out that the difference in sensitivity is due simply to a difference in the degree of activation of the alleles.

What about the role of EAT-16? The assumption for the following test is that if the G-αq protein is the sole component interacting with the compound, then the presence or absence of the RGS protein should not affect resistance/sensitivity to the compound. Conversely, if presence of the RGS protein is critical for compound activity, removing it (by mutation) should confer resistance regardless of the status of the G-αq subunit. Therefore, a double mutant strain containing the compound-sensitive *egl-30(pk931)* allele and the RGS protein loss-of-function *eat-16(ad702)* allele was constructed and tested for small-molecule sensitivity. The *eat-16(ad702); egl-30(pk931)* double mutant strain was found to be completely resistant to BMS-192364 (Figure 4C), like the *eat-16(ad702)* single mutant, implicating EAT-16 in compound action. Overall, these results are consistent with a model in which both the G-αq and RGS proteins interact with the compound.

### Chemical Genetic and Biochemical Tests of the G-α Antagonist Model in Mammalian Cells

To investigate the G-α antagonist model in mammalian cells utilizing a better characterized G-αq allele, we overexpressed the mammalian G-αq-G188S mutant protein in Hek-293 cells (construct courtesy of the Guthrie Research Institute). The G-αq-G188S mutant protein requires ligand stimulation, but is known to be resistant to subsequent deactivation by RGS proteins, thus behaving as a constitutive signaling molecule [12]. However, overexpression of G-αq-G188S did not suppress the inhibitory effect of BMS-192364 upon carbachol-stimulated calcium flux (Figure 7C). This result (similar to the G-αq experiment in C. elegans above), also fails to support a model where compound directly antagonizes G-αq.

Finally, we tested the compound's direct biochemical action on wild-type G-αq. We quantified binding of a radio-labeled nonhydrolysable substrate (GTP-γS) to the G-α protein downstream of the histamine H1 receptor (which is G-αq coupled) [47] and the muscarinic receptor M4 (which is G-αi/G-αo coupled) [48]. BMS-192364 displayed no significant activity in either assay, when performed in either agonist or antagonist mode (unpublished data). These results suggest that BMS-192364 does not directly affect GDP/GTP exchange at G-αq or G-αi/o.

## Discussion

Here we investigate the mechanism of action for a novel class of small molecules that are effective in assays for bladder capacity and spontaneous bladder muscle contraction. We have utilized C. elegans as a model system to investigate this mechanism. Treatment of *C. elegans* with the compounds produced a neuromuscular phenotype, i.e., decreased egg laying, that correlated with the therapeutic activity in the small-molecule series. The phenotype was used as the basis for candidate gene analysis, and for a genetic resistance screen. Strikingly, both approaches uncovered components of the GPCR signaling pathway, a proven therapeutic target in UI disease. The genetic screen uncovered dominant mutations in two proteins (G-αq and RGS) that form an important regulatory complex.

The results of subsequent genetic and biochemical analyses were not consistent with models in which the compound acts solely on either the G-α or the RGS protein (Figure 8, models 1 and 2). However, the results are consistent with models in which the small molecule affects the RGS/G-αq complex and results in the termination of GPCR signaling, as well as alternative models involving adaptation responses to calcium signals. If the small molecule increased the affinity of the RGS protein for the G-αq transition state, there would be an increase in the rate of RGS protein GAP activity (Figure 8, model 3). Alternatively, the small molecule could stabilize RGS/G-α in an inactive complex, thus effectively acting as a noncompetitive inhibitor of the G-αq signal (Figure 8, model 4). In either case, the small molecule could interact with both the RGS protein and G-αq during the GTPase transition state. Both of these mechanisms require the physical presence of RGS protein for compound activity, and are consistent with all of the available data.

In considering these alternative models based on interaction with the RGS/G-αq complex, we were struck by reports concerning the small molecule brefeldin A and its effect on the ARF1/Sec7 complex. The relationship between ARF1 and Sec7 is partially analogous to that of G-αq and RGS: *Arf1* is a small GTPase of the Ras family, while Sec7 physically interacts with Arfs to catalyze guanine nucleotide exchange (GEF activity), thereby affecting downstream signal transduction. The limitation to this analogy is that Sec7 has GEF activity whereas RGS proteins have GAP activity, thus they represent different stages of the guanine nucleotide cycle. However, in both cases it is thought that the reactions involving guanine nucleotide require formation of docking intermediates with the GTPase. Brefeldin A binds to and stabilizes the *Arf1*-GDP-Sec7 domain protein complex (the first intermediate on the nucleotide exchange reaction pathway). It is thought that brefeldin A prevents an important region of *Arf1*-GDP from reorganizing. Brefeldin A activity is also sensitive to mutations in both Sec7 and *Arf1*. Thus far our efforts to prove a direct effect of the compounds on an RGS/G-Protein complex have not been successful. These efforts have greatly been hampered by the difficulty in obtaining purified, active G-αq protein, and the very limited solubility of the compounds in immunoprecipitation experiments.

In addition to the models above, others remain possible. RGS proteins have been shown to interact with proteins other than G-proteins. Alternative (or additional) interaction partners include kinases, G-β5, and GPCRs. Recent unpublished data from our group suggests that these componds have a disruptive effect on an RGS protein/GPCR interaction within cells (KF, personal communication). Disruption of this interaction could conceivably result in additional free RGS. However, the significance of this disruption, and the consequences of it in the context of G-protein signaling, remains to be further investigated.

These studies have uncovered a novel set of mutants that will provide significant insight into G-α and RGS protein function. Our analysis of the compound-resistant G-α M244I allele using a yeast assay indicates that it causes increased sensitivity to continued presence of ligand (hypo-adaptation). While hyper-adaptation alleles that affect the same region of the protein have been uncovered, we believe this is the first hypo-adaptation allele to be described for G-α. Hypo-adaptation alleles of the yeast G-β protein Ste4 have been identified as suppressors of a *GPA1* hyper-adaptation allele [44]. Since hypo- and hyper-adaption mutations of heterotrimeric G-protein components affect their sensitivity to GPCR agonist–antagonist activity in genetic model systems, variations of these proteins in patient populations may be related to differential responses to GPCR modifying therapies.

It is also noteworthy that we identified a dominant resistant mutation in EAT-16 (E158K) that did not affect the well-defined RGS, GGL, or DEP domains (Figure 4B). Existing crystal structures of RGS complexes are limited to the RGS domain itself, and the role of other regions of the protein is not as clear. Previous work has implicated the region of EAT-16 containing the E158K mutation in determining specificity of the RGS/G-α interaction, and in the binding and stabilizing of RGS by G-β5 [24]. It is possible that the EAT-16 E158K mutation results in an inability to bind G-β5 and consequent protein instability, leading to the observed RGS loss-of-function phenotype.

In this work, we have used an unusual combination of technologies: small-molecule screens, genetic analysis in two model systems, and biochemical assays. The relevance of the pathway and targets suggested by the model system genetics was validated by assays on mammalian systems. While the exact mechanism of action of these compounds remains under investigation, it is certain that these small molecules have a unique action downstream of muscarinic GPCRs, and that they function by limiting G-α signaling. The discovery of such compounds, as well as the unique RGS and G-α mutations uncovered here, has implications for GPCR activity modifying therapies. This work also supports the notion that small molecules affecting pathways downstream of GPCR function in novel ways, and could represent potential new therapies or biomarkers for diseases characterized by inappropriate activation of GPCR signaling.

## Materials and Methods

All experiments that involved collecting and processing animal tissue samples were performed using animal test methods approved by the Bristol-Myers Squibb Institutional Animal Care and Use Committee, and in accordance with the Guide for the Care and Use of Laboratory Animals as adopted and promulgated by the National Institutes of Health.

### Rat whole bladder ex vivo model.

The model used for functional experiments was a modified version of that described in 1986 by Malkowicz et al. [49]. Briefly, a female rat (250–350 g) (Harlan Sprague Dawley, Indianapolis, Indiana, United States) was sacrificed by decapitation. The bladder was excised, cleaned of connective tissue, and the ureters were tied. The bladder was catheterized at the urethral opening and mounted in a 50-ml organ bath containing normal Kreb's buffer (composition in mM: NaCl 118.4, KCl 4.7, KH_2_PO_4_ 1.2, MgSO_4_ 1.3, CaCl_2_ 1.8, Glucose 10.1, NaHCO_3_ 25, gassed with 95% O_2_/5% CO_2_, 37 °C). The bladder was infused with normal Kreb's buffer at 0.05 ml/min for 30 min to a maximum volume of 1.5 ml. The pressure developed in the bladder during the infusion was measured using a pressure transducer (model P23XL, Ohmeda, Norcross, Georgia, United States) and an *AcKnowledge* data acquisition system (MP100WS, Biopac Systems, Goleta, California, United States). At the end of infusion the bladder was allowed to empty and the volume of “spontaneous” bladder emptying was measured. Carbachol (1 and 10 μM) was added to the bath to induce complete bladder emptying (unpublished data). Bladders were subjected to a series of three infusions: 1) a “conditioning” infusion with normal Krebs followed by a 1-h recovery period; 2) a “control” infusion followed by 1-h incubation with BMS-195270 or vehicle (in paired controlled experiments); and 3) a final infusion in the presence of BMS-195270 or vehicle. Statistical analysis of the data was performed using an unpaired *t*-test; *p* < 0.05 was considered significant (GraphPad InStat, version 3.05, GraphPad Software, San Diego, California, United States).

### Ca++ flux assays.

HEK293 cells or primary human bladder cells were seeded at a density of 5 × 10^4^ per well on Poly-D-lysine–coated 96-well white/clear Biocoat microtiter plates (Becton-Dickinson, Bedford, Massachusetts, United States) overnight in DMEM +10% FBS. Cells were washed 3× in 100-uL Kreb's buffer (NaCl 118 mM, KCl 4.7 mM, MgSO_4_ 1.2 mM, KH_2_pO_4_ 1.2 mM, NaHCO_3_ 4.2 mM, D-Glucose 11.7 mM, CaCl_2_ 1.3 mM, Hepes [pH 7.4] 10 mM). Cells were equilibrated (“loaded”) with 4 uM Fluo-4 (Molecular Probes, Eugene, Oregon, United States) in Kreb's buffer containing 0.08% (wt/vol) Pluronic F-127 and 0.25 mM sulfinpyrazone for 1 h with the plate covered in foil. Cells were washed 2× in 100-uL Kreb's buffer containing 0.5% BSA and 0.25 mM sulfinpyrazone. The BMS compound stocks were made up in DMSO and diluted to working concentrations in Kreb's buffer containing 0.5% BSA and 0.25 mM sulfinpyrazone. 100uL of either the test compound or vehicle was added to the wells and the plate incubated for 15 min at room temperature. The [Ca^2+^]_i_ signal was evoked by addition of histamine or carbachol at the concentrations indicated in figure legends. Two detection systems were used and yielded similar results. In system one, Fluo-4 was excited at 488 nm and fluorescence emission at 510 nm was determined simultaneously from multiple wells in a time-resolved mode (1 Hz frequency) using a fluorometric plate reader (FLIPR, Molecular Devices, Sunnyvale, California, United States). Relative fluorescence intensity was used as indication of evoked [Ca^2+^]_i_ signal. Data acquisition and preliminary analysis was performed using FLIPR software (Molecular Devices). Alternatively, Fluo-4 was measured with the 488 excitation/535 emission filter set on a well-by-well basis using a Wallac Victor2 fluorometer (PerkinElmer, Boston, Massachusetts, United States). In both systems, a baseline fluorescence was measured at each second for 5 s, followed by agonist injection. Subsequent readings were taken at each second up to 60 s and then every 2.5 s up to 75 s. The EC50s were obtained and graphs generated using GraphPad Prism software (GraphPad Software). The EC50 for BMS 195270 in these assays was 2 μM and for BMS-192364 was 9 μM.

For experiments with niguldipine pretreatment, cells were loaded with Fluo-4 dye as described above. Treatment with 100 μM niguldipine (IC50 75 nM, Sigma-Aldrich, St. Louis, Missouri, United States) was initiated 15 min prior to addition of the small molecules under test. Control wells received an equal concentration of vehicle. The cells were then treated with either test small molecules or vehicle for 15 min, followed by stimulation with 100 μM carbachol.

For experiments with pertussis toxin, cells were pretreated for 24 h in complete media with 150 ng/ml pertussis toxin (Calbiochem, San Diego, California, United States) or a vehicle control. Cells were then washed 3× in media and loaded with Fluo-4 as described above. Cells were then treated with either test small molecules or vehicle for 15 min followed by stimulation with 100 μM carbachol.

### Radio-ligand binding assays.

All radio-ligand–binding and GTP-γS assays were carried out by MDS Pharma Services, Taiwan. M1–M5 muscarinic receptor assays were carried out essentially as in [28,29]: recombinant M1–M5 were expressed in CHO cells and were used in a modified Tris-HCL buffer (pH 7.4). A 16-mg aliquot was incubated with 0.8 nM {^3^H}N-methylscopolamine for 120 min at 25 °C. Nonspecific binding was estimated in the presence of 1 μM atropine. Membranes were filtered and washed. The filters were counted to determine the specifically bound ^3^H. BMS compounds were tested at 10 μM.

Calcium channel–binding assays were carried out essentially as in [30,34]. Briefly, frontal lobe brains of male Wistar-derived rats weighing 175 g were used to prepare N- or L-type calcium channels in modified Tris-Hcl buffer (pH 7.4). A 40-ug aliquot was incubated with either 10 pm {^125^I}ω-conotoxin GVIA (N-type channel inhibitor) for 30 min at 4 °C, or a 0.5 mg aliquot was incubated with 2 nM {^3^H} diltiazem for 180 min at 4 °C. Nonspecific binding was estimated in the presence of either 100nm ω-conotoxin GVIA or 10 μM diltiazem. Membranes in each case are filtered and washed with the filters subsequently counted for radioisotope. BMS compounds were screened at 10 μM.

M4 and H1 GTP-γS assays were carried out under standard conditions in both agonist and antagonist modes [47,48]. Briefly, human muscarinic receptor M4 was expressed in Sf9 cells. Test compound was pre-incubated with 0.2 mg/ml membranes and 3 μM GDP for 20 min at 25 °C in modified HEPES buffer (pH 7.4). SPA beads were added for another 60 min at 30 °C. The reaction was then initiated by addition of 0.3 nM {^35^S}GTP-γS for 15 min. BMS compounds were tested at 10, 1, 0.1, and 0.001 μM for their ability to increase GTP-γS binding relative to 10 μM McN-A-343 (indicating possible agonist activity), or to inhibit an increase in binding rendered by 1 μM dopamine. For histamine H1 assays, the receptor was expressed in CHO cells. BMS compounds were pre-incubated with 0.022 mg/ml receptor and 1 μM GDP in modified HEPES buffer (pH 7.4) for 20 min at 25 °C, SPA beads were added for another 60 min at 30 °C. The reaction was then initiated by addition of 0.3M {^35^S}GTP-γS for 30 min. BMS compounds were tested at 10, 1, 0.1, and 0.001 μM for their ability to increase or decrease GTP-γS binding relative to 10 μM histamine, indicating possible agonist or antagonist activity.

### Phenotypic and genetic analysis of *C. elegans.*

C. elegans strains were cultured and maintained according to standard procedures. All strains were assayed at 20 °C unless otherwise indicated. Sequence analysis showed that the eat-16*(ad702)* allele carries an AG → AA mutation in the splice acceptor site before the fourth exon, which results in early termination before the RGS domain that interacts with G-αq. This allele shows no additional phenotypes when placed opposite a chromosomal deficiency that deletes the entire RGS protein coding region; i.e., it behaves as a null allele for *eat-16*.

### Small-molecule treatment of *C. elegans.*

Treatment of C. elegans with the small molecules was conducted as follows: a solution of the small molecule in DMSO was mixed with a slurry of killed bacteria (strain OP50, taken through multiple freeze–thaw cycles) to twice the desired final concentration. Adult wild-type or mutant hermaphrodites (Bristol N2 strain) were collected in standard M9 media. Worms were mixed with the small-molecule/bacteria slurry in a 1:1 ratio, and plated on peptone-free NGM plates. The final DMSO concentration did not exceed 1%. At 1, 2, 4, 8, and 16 h of treatment, worms were observed and assessed for behavioral and visible defects.

For egg-laying assays, adults were treated overnight with the small molecule. Approximately thirty animals were then loaded onto agar pads made on glass slides, and examined under Nomarski optics. Animals were scored as egg-laying defective if they contained developing (“comma stage”) embryos.

### EMS mutagenesis/screening of *C. elegans.*

EMS mutagenesis was conducted according to standard procedures [50]. Briefly, Bristol N2 hermaphrodites of L4 stage were treated with 0.25% EMS (Sigma-Aldrich) in M9 for 4 h at 20 °C. Worms were washed 4× in M9 media, and plated onto seeded NGM plates. Staged collections were taken of the F1 generation, and these were plated onto NGM plates at either 20 °C or 15 °C. Staged collections of the F2 generation were plated onto NGM plates and allowed to grow until adulthood. These adults were then collected and treated with small molecule. After an overnight treatment with the test compounds, animals that were not visibly egg-laying defective were isolated and retested for resistance to small molecule.

### Characterization of alleles from C. elegans mutants.

Mutant hermaphrodites were crossed to males of the polymorphic strain CB4856 (Hawaiian isolate). Recombinant homozygous mutants in the F2 generation were selected by their visible phenotype and assayed for SNPs. Genotyping of SNP markers was performed using standard methods and employed SNPs identified either through the Washington University SNP project or privately at Exelixis. The map data generated was as follows:

*egl-30(ep271)*: ChrI; −17.9 cM to −5.53 cM (~1700 kb)

*ep272*: Chr I; −10.31 cM to −8.76 cM (~150 kb)

*eat-16(ep273)*: ChrI; 2.63 cM to 4.4 cM (~2 Mb)

*goa-1(ep275)*: ChrI; 2.03 cM to 3.74 cM (~2.6 Mb)

For the *eat-16(ep273), egl-30(ep271),* and *goa-1(ep275)* alleles, the identity of the mutant gene was confirmed by sequence analysis of PCR products templates upon genomic DNA from the mutant strains. Sequencing of PCR products was performed according to manufacturer's instructions (Applied Biosystems, Foster City, California, United States).

### Construction and analysis of yeast strains.

Centromeric vectors from the pRS series [43] were used to provide *GPA1* and *SST2* gene functions as follows. A 2.8-kb PCR fragment containing the wild-type yeast *GPA1* gene and promoter was amplified from genomic DNA using primers CGGGATCCAAGAGCCCAAGTATGTAA and CGGGATCCTCATATAATACCAATTTTT and cloned into pCR.TOPO2.1 (Invitrogen, Carlsbad, California, United States). A *Bam*HI fragment containing the amplicon was moved to pJC72 (pRS415 with the *PHO5* transcription terminator) to create plasmid pJC73. Site-directed mutagenesis of pJC73 using the Quikchange system (Stratagene, La Jolla, California, United States) with the oligonucleotide primers GGATGAAAGAGTGAACAGAATTCATGAATCAATAATGCTATTTG and CAAATAGCATTATTGATTCATGAATTCTGTTCACTCTTTCATCC generated plasmid pJC74, containing the *gpa1-M362I* substitution. The full *GPA1* gene sequence was confirmed in pJC73 and pJC74. To provide Sst2 function, a 2-kb amplicon containing the coding sequence was generated from genomic DNA using primers GAGGATCCATGGTGGATAAAAATAGGACG and CGGTCGACTTAGCACTTTTCTTGGATTTC. The amplicon was cloned into p416-TEF (pRS416 with the *TEF1* promoter sequence and the *CYC1* terminator) to create plasmid pJC156.

Strain Y87 *(MAT*a *gpa1 sst2 leu2 ura3 his3)* was isolated from a meiotic tetrad that showed 2:2 segregation of G418-resistance from a cross between RG6055 and RG16602 (Research Genetics, Huntsville, Alabama, United States). Y87 has the slow growth phenotype expected of a *gpa1* mutant; this phenotype was complemented following transformation with pJC73 or pJC74. For halo assays, a suspension of yeast cells (OD600 0.3) was poured onto solid yeast growth medium and the excess liquid drained. Medium was synthetic complete minus leucine for pJC73 or pJC74; synthetic complete minus uracil and leucine for pJC73 or pJC74 with pJC156. 10 μl of a 2-mg/ml solution of α-factor in water was placed on a 7-mm glass fiber disc (Schleicher and Schuell Bioscience, Keene, New Hampshire, United States) at the center of the plate. Plates were incubated at 30 °C for 24 h before photography.

## Abbreviations

BMS: Bristol-Myers Squibb
Eat: arrested pharyngeal pumping
Egl-c: egg-laying constitutive
EMS: ethyl methanesulfonate
G-αq: G-protein alpha subunit
GAPs: GTPase activating proteins
*(gf)*: gain-of-function
Gpa1: (the G-protein alpha subunit)
GPCR: G-protein coupled receptor
GTP: guanosine triphosphate
*(lf)*: loss-of-function
M3: muscarinic receptor type 3
PKC: protein kinase C
RGS: regulators of G-protein signaling
SNP: single nucleotide polymorphism
UI: urinary incontinence
Unc: uncoordinated motion
